# Fully automated 3D body composition analysis and its association with overall survival in head and neck squamous cell carcinoma patients

**DOI:** 10.3389/fonc.2023.1176425

**Published:** 2023-10-19

**Authors:** Miłosz Rozynek, Daniel Gut, Iwona Kucybała, Ewa Strzałkowska-Kominiak, Zbisław Tabor, Andrzej Urbanik, Stanisław Kłęk, Wadim Wojciechowski

**Affiliations:** ^1^ Department of Radiology, Jagiellonian University Medical College, Krakow, Poland; ^2^ Department of Biocybernetics and Biomedical Engineering, AGH University of Science and Technology, Krakow, Poland; ^3^ Department of Statistics, Universidad Carlos III de Madrid, Madrid, Spain; ^4^ Surgical Oncology Clinic, Maria Skłodowska-Curie National Cancer Institute, Krakow, Poland

**Keywords:** body composition, artificial intelligence, head and neck cancer, overall survival, squamous cell carcinoma

## Abstract

**Objectives:**

We developed a method for a fully automated deep-learning segmentation of tissues to investigate if 3D body composition measurements are significant for survival of Head and Neck Squamous Cell Carcinoma (HNSCC) patients.

**Methods:**

3D segmentation of tissues including spine, spine muscles, abdominal muscles, subcutaneous adipose tissue (SAT), visceral adipose tissue (VAT), and internal organs within volumetric region limited by L1 and L5 levels was accomplished using deep convolutional segmentation architecture - U-net implemented in a nnUnet framework. It was trained on separate dataset of 560 single-channel CT slices and used for 3D segmentation of pre-radiotherapy (Pre-RT) and post-radiotherapy (Post-RT) whole body PET/CT or abdominal CT scans of 215 HNSCC patients. Percentages of tissues were used for overall survival analysis using Cox proportional hazard (PH) model.

**Results:**

Our deep learning model successfully segmented all mentioned tissues with Dice’s coefficient exceeding 0.95. The 3D measurements including difference between Pre-RT and post-RT abdomen and spine muscles percentage, difference between Pre-RT and post-RT VAT percentage and sum of Pre-RT abdomen and spine muscles percentage together with BMI and Cancer Site were selected and significant at the level of 5% for the overall survival. Aside from Cancer Site, the lowest hazard ratio (HR) value (HR, 0.7527; 95% CI, 0.6487-0.8735; p = 0.000183) was observed for the difference between Pre-RT and post-RT abdomen and spine muscles percentage.

**Conclusion:**

Fully automated 3D quantitative measurements of body composition are significant for overall survival in Head and Neck Squamous Cell Carcinoma patients.

## 1 Introduction

Head and neck squamous cell carcinoma (HNSCC) had 890,000 new cases and 450,000 new deaths in 2018, placing itself as the 6^th^ most common cancer worldwide (1). It arises from the squamous epithelium of the oral cavity, oropharynx, larynx, and hypopharynx (2) and due to its anatomical location and clinical symptoms such as chronic pain of the throat, nonhealing ulcers, and odynophagia, it can cause a decrease in proper food intake. Additionally, as a consequence of the treatment schemas currently used, patients struggling with this disease could also suffer from the toxicity of radio- and chemotherapy (3, 4). Furthermore, cancer catabolic properties and lifestyle risk factors such as high alcohol consumption and nicotine dependence may also contribute to an increased risk of malnutrition in these patients (5).

Consequently, their muscle and adipose tissue can potentially have disturbed proportions and properties. It is an important issue that we should look at, considering that recent studies suggest that body composition (BC) has a significant impact on different clinical outcomes in oncology (6). There are a few ways to obtain information about the BC of the patient, from these methods cross-sectional imaging techniques including CT and magnetic resonance imaging are considered the gold standard (7). From those two imaging modalities, CT is more commonly used, especially in oncology. It is a routine diagnostic tool; therefore, almost every oncological patient has performed at least one and sometimes even more CT scans, in the case of patients with HNSCC PET/CT is the recommended diagnostic modality in the evaluation of distant metastases (4, 8). However, body composition analysis, although potentially important for a complete diagnosis, is not routinely used by clinicians. This is due to the fact that currently such a measurement needs to be performed by segmenting numerous cross-sectional images, which is a tedious and time-consuming task. The answer to this problem could be found in artificial intelligence, which can help radiologists perform automated and standardized body composition measurements (9).

In our study, we wanted to develop a Fully Automated Method for 3D segmentation of tissues within the volumetric body region limited by L1 and L5 levels and verify if those measurements are significant for the overall survival of patients treated due to head and neck squamous cell carcinoma. We also wanted to find out if Fully Automated 3D measurements of body composition are a better predictor of overall survival than manual 2D cross-sectional measurements derived from L3 level.

## 2 Materials and methods

### 2.1 Materials

We used two separate datasets: the first dataset was used to develop a deep learning model capable of fully automated 3D abdominal tissue segmentation. The other, on which our model was used to obtain quantitative measurements of body composition, was made up of imaging and clinical data of HNSCC patients.

#### 2.1.1 Head and neck squamous cell carcinoma patients dataset

This dataset was retrieved from the Head and Neck Cancer CT atlas available in “The Cancer Imaging Archive” (TCIA) (10–12). TCIA provides anonymized data with consent obtained and ethical approval ensured by source institutions. It consists of clinical and imaging data of HNSCC patients treated with curative-intent radiotherapy (n=215). They were selected from the group of 2840 HNSCC patients hospitalized at MD Anderson Cancer Center from October 1, 2003, to August 31, 2013. The dataset comprised 215 HNSCC patients, 84,7% were male, mean age was 57,21 ± 9,79 years. Most patients (62,36%) had a smoking history. Each patient had histologically confirmed squamous cell carcinoma, the most common primary cancer site was the oropharynx (72,6%). 114 of 215 patients received a feeding tube. 59,1% received concurrent chemotherapy; in 98,4% of the cases, it was platinum-based. All patients were treated with radiotherapy (RT), the mean total dose was 68,73 ± 2,71 Gy. 212 patients had whole body PET/CT done before and after radiotherapy, in the case of 3 patients, an abdominal CT scan was used instead. GE Medical Systems’ Discovery RX, Discovery ST, and Discovery STE hybrid PET/CT scanners were utilized to acquire PET/CT scans subsequent to the intravenous administration of 18F-labeled fluorodeoxyglucose. CT scans, encompassing the abdominal area, were conducted using GE Medical Systems LightSpeed or Discovery CT750HD scanners. All the details for each examination, such as image type, date, study description, used scanner, and software specifications, can be found on the TCIA website (10). The summary of patient data can be found in **Table 1**. Aside from standard clinical information authors of the database also provided us with their manual 2D cross-sectional body composition measurements: Pre-RT L3 Skeletal Muscle Index (cm2/m2), Post-RT L3 Skeletal Muscle Index (cm2/m2), Pre-RT L3 Adiposity Index (cm2/m2) and Post-RT L3 Adiposity Index (cm2/m2). These indices were calculated by diving the cross-sectional area of selected tissue at the L3 level by the height of the patient. We used those 2D measurements to compare them with our fully automated 3D body composition measurements.

**Table 1:** Summary of patients and treatment characteristics.

Summary of patients data:	
Total number of patients	215 (100%)
Male	182 (84,7%)
Female	33 (15,3%)
Age (years)	57,21 (± 9,79)
Height (m)	1,74 (± 0,09)
Body weight at the start of radiotherapy (kg)	85,61(± 18,15)
BMI at the start of radiotherapy	28,37 (± 5,51)
Smoking History	136 (63,26%)
Current Smoker	70 (32,56%)
Received Feeding Tube	114 (53,02%)
Histology:	
Squamous cell carcinoma	215 (100%)
Stage (AJCC 7th edition):	
IVB	19 (8,8%)
IVA	156 (72,6%)
III	31 (14,4%)
II	5 (2,3%)
I	4 (1,9%)
Site:	
Oropharynx	156 (72,6%)
Glottis	24 (11,2%)
Hypopharynx	12 (5,6%)
Oral Cavity	8 (3,7%)
Unknown	6 (2,8%)
Nasopharynx	6 (2,8%)
Sinus	3 (1,4%)
Chemotherapy:	
Yes	127 (59,1%)
No	88 (40,9%)
Surgery:	
Yes	69 (32,09%)
No	146 (67,91%)
Radiotherapy:	
Radiotherapy total dose (Gy)	68,73 (± 2,71)

AJCC, American Joint Committee on Cancer.

#### 2.1.2 Model training and testing dataset

The training and testing dataset comprised 140 CT examinations performed with the use of a helical 80-row CT scanner Aquilion PRIME 80 (Toshiba America Medical System, Irvine, CA, USA). The pixel size of the images was equal to 0.74 mm, while the slice thickness was equal to 5 mm. The images were coded with a contrast resolution equal to 2 bytes but, in accordance with the DICOM standard, only 12 bits were used to encode signal values with Hounsfield units. Further details concerning the dataset used to develop our model can be found elsewhere (13). From these 140 3D abdominal CT examinations, 560 2D slices were manually selected at levels corresponding to the centers of the lumbar vertebral bodies L1 to L5. The location of L1 and L5 slices within each CT examination was also recorded and used as a reference when creating a model for detecting an abdominal part of a CT image.

### 2.2 Methods

#### 2.2.1 Automated assessment of tissues’ volumes

To develop a fully automated tissues volumes assessment method, we divided our task into two separate problems: detection of the volume of interest and segmentation of tissues of interest.

For detecting the volume of interest, the 3D CT examinations described in section 2.1.2 were split into 2D axial slices. All slices containing part of the lungs or parts of the pelvic bone were assigned a negative label, while the remaining slices were assigned a positive label. The 2D slices together with their labels were used to train an algorithm that detects volumes of interest.

The detection of the volume of interest (VOI) was accomplished using a deep convolution network. The detection problem was reformulated as a classification problem. An Inception-based classification network was trained to distinguish between positive and negative slices using the custom dataset described in the previous section. The volume of interest was defined as the range of axial slices between the lowest and the highest positive slice. The set of all 140 CT images was split into 5 folds and five models were trained until convergence using five-fold cross validation. The models that achieved the best performance during training on the validation sets were used on TCIA dataset. The classification accuracy was measured in terms of the distance between the respective borders of the volumes of interest in a reference (manual) selection and in automatically detected volumes of interest. In addition, we also calculated the common measures of classification quality: sensitivity, specificity, and accuracy by calculating macro averages of five confusion matrices for the five validation sets for the classification task.

For segmentation purposes, the boundaries of the spine, spine muscles, abdominal muscles, subcutaneous adipose tissue (SAT), and visceral adipose tissue (VAT) were manually outlined and served as the ground truth. Internal organs were also included as separate regions consisting of all pixels that were within the body cross section but were not included within the aforementioned classes. From the data set described in section 2.1.2 - 560 single-channel CT slices were split as follows: 420 images were randomly assigned to a training subset while the remaining 140 images were assigned to the testing subset.

The segmentation of abdominal tissues was done with a deep convolutional segmentation model - U-Net network (14). As shown in the study of Weston et al. (15) the U-Net based segmentation of abdominal tissues achieves an accuracy exceeding 95% in terms of Dice’s coefficient. We have used the U-Net model implemented in the nnUNet framework which includes several features improving the performance of the trained models like architecture to data adaptation, deep supervision, or learning rate strategy which makes this segmentation framework superior with respect to other segmentation models (16). Furthermore, the automated segmentation pipeline incorporates resampling and normalization methods, facilitating the standardization of images. This integration helps mitigate biases arising from variations in scanners and image qualities. We have trained the 2D version of U-Net – the volume of interest was split into 2D axial slices which were subsequently used to train and test the 2D U-Net network. According to recently published study Classical U-Net architecture presents themself as the most favorable option, which strikes a balance between accuracy and computational efficiency, making it appropriate choice for real-world applications such as automated body composition analysis (17). Both training and testing were run using default nnUNet settings. The training of segmentation was run until convergence on a training set. Five-fold cross-validation was used to train five models that were used as an ensemble at prediction time. During each fold training, the model which achieves the best performance on a validation set was saved and used at the prediction time.

The accuracy of segmentation was measured in terms of two standard segmentation quality metrics: Dice’s coefficient (DC) and Hausdorff distance (HD) with DC (HD) equal to 1 (0) for automated segmentation being the same as reference segmentation.

**Figure 1:**
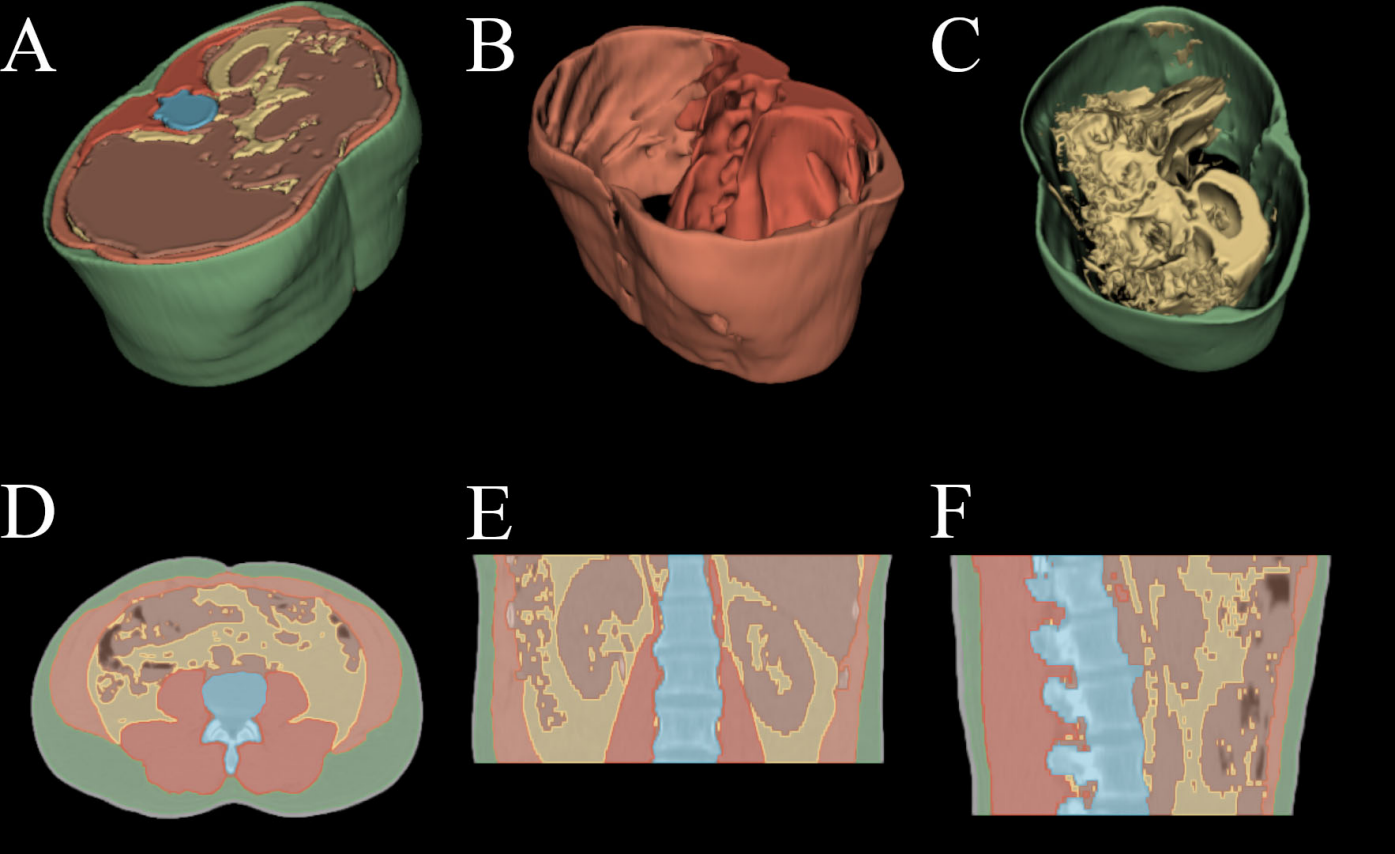
Automated segmentation applied to HNSCC patient PET/CT examination. **(A)** 3D render of all above mentioned tissues. **(B)** 3D render of spine and abdominal muscles. **(C)** 3D render of subcutaneous and visceral adipose tissue. **(D)** axial slice with overlayed segmentation mask. **(E)** coronal slice with overlayed segmentation mask. **(F)** sagittal slice with overlayed segmentation mask.

After training and testing on the custom dataset the deep segmentation U-Net model and the classification model were applied slice-by-slice to the Cancer Imaging Archive dataset described in section 2.1.1. A sample image of our automated segmentation applied to the TCIA dataset is shown in **Figure 1**.

#### 2.2.2 Survival analysis methods

We conducted a multivariate analysis using Cox proportional hazard (PH) model to check whether the parameters assessed by automated 3D body composition measurements and their changes are significant for overall survival time. For this, we considered all the important clinical data together with the PET/CT based body composition measurements. In particular, we included to the model the following variables: sex (m/f), age (cont.), height (cont.), stage (I-III/IV), T stage (T1-3/T4), N stage (N0/N1-3), Cancer Site (Oropharynx/Other site), Smoking History (Never/Ever), Current Smoker (Yes/No), Surgery (Yes/No), Concurrent chemoradiotherapy (Yes/No), Induction Chemotherapy (Yes/No), Received Feeding Tube (Yes/No), RT Total Dose (cont.) together with BMI, 2D manual measurements and our automated 3D measurements. All body composition measurements were collected both before and after the Radiotherapy. Furthermore, we reduced the number of categorical variables to those significant at the level of 10% in the univariate analysis (log-rank test). Although HPV-negative and HPV-positive carcinomas differ at the molecular level (4) in our data set, there were only 47 patients with known HPV status, of which 33 were positive. In the previously mentioned log-rank test differences in survival time between groups: HPV positive and HPV negative were not significant. Moreover, the preliminary correlation analysis showed a relatively strong dependence between SAT/VAT pre-treatment measurements and BMI measured before treatment. Hence, those two variables were excluded from our study. Additionally, the measurement of the spinal and abdominal muscles was only considered as a sum.

Furthermore, for 77 patients we observe the complete survival time, which gives us around 64% of censoring. We fitted the proportional hazard (PH) model where, along with patients characteristics, the Pre RT measurements together with the differences between pre RT and post RT measurements were taken into account. The statistical analysis was conducted using the coxph() routine from open-source statistical software R. We selected the final set of covariates using Akaike Information Criterium (AIC). We verified the PH assumption with the Schoenfeld test and checked for outliers and influential observations with deviance residuals and dfbetas, respectively. One influential observation was removed.

## 3 Results

### 3.1 Automated segmentation results

The mean shift between the bottom slices in manual and automated VOIs was equal to -0.6 ± 1.65 slices. The mean shift between the top slices in manual and automated VOIs was equal to 0.2 ± 1.0 slices. Additionally, sensitivity, specificity, and accuracy were equal to, respectively, 96.1%, 96.2%, and 96.2%. The detection of the bottom slices failed in three cases, the shift between the bottom slices in manual and automated VOIs was larger than 20 slices.

The results of testing the automated segmentation algorithm are presented in **Table 2**. The median values of Dice’s coefficient (0.95 and more) indicate that the overlap area between automated and manual segmentation is not less than 95% of the total area of the tissue. The median values of Hausdorff distance are greater than 6 voxels, which means that the relative shift of the boundaries of manual and automated segmentation can be as large as demonstrated in **Table 2**. However, because the values of Dice’s coefficient are high, these high values of Hausdorff distance are likely related to some small segmentation artefacts or anisotropic voxel size.

**Table 2:** The results of testing the quality of automated segmentation: values of Dice’s coefficient and Hausdorff distance (median and interquartile range) for the six segmented abdominal tissues.

Tissue	Dice’s coefficient	Hausdorff distance [voxels]
Subcutaneous adipose tissue	0.99 (0.01)	8.0 (7.2)
Visceral adipose tissue	0.97 (0.03)	29.2 (24.5)
Internal organs	0.96 (0.01)	35.0 (18.9)
Spine	0.97 (0.01)	3.0 (2.8)
Spine muscles	0.96 (0.01)	5.8 (3.5)
Abdominal muscles	0.95 (0.02)	7.8 (7.2)

### 3.2 Automated 3D body composition quantitative analysis results

The VOI detection and tissue segmentation models were applied to TCIA dataset described in section 2.1.1. Results were visually verified after which it was confirmed that they were of satisfactory diagnostic quality. The volumes of the tissues of interest within the VOI were then calculated from the results of the segmentation. We calculated both the average tissue volume within VOI (total tissue volume divided by the number of axial slices within the VOI) and the volume fraction of tissues (ratio of tissue volume to the total volume of all tissues) which percentage values were used for survival analysis. The summary of those measurements can be found in **Table 3**.

**Table 3:** The mean and standard deviation of body measurements pre RT, and post RT and their differences.

Variables	Pre RT measurements	Post RT measurements	Difference in measurements
**SAT (%)**	20.82 (8.52)	18.37 (8.77)	-2.48 (3.91)
**VAT (%)**	23.16 (8.62)	18.87 (8.47)	-4.28 (4.06)
**Spine Muscles (%)**	10.00 (1.91)	10.36 (2.05)	0.38 (1.14)
**Abdomen Muscles (%)**	10.40 (1.83)	10.26 (1.89)	-0.13 (1.14)

### 3.3 Survival analysis results

**Figure 2:**
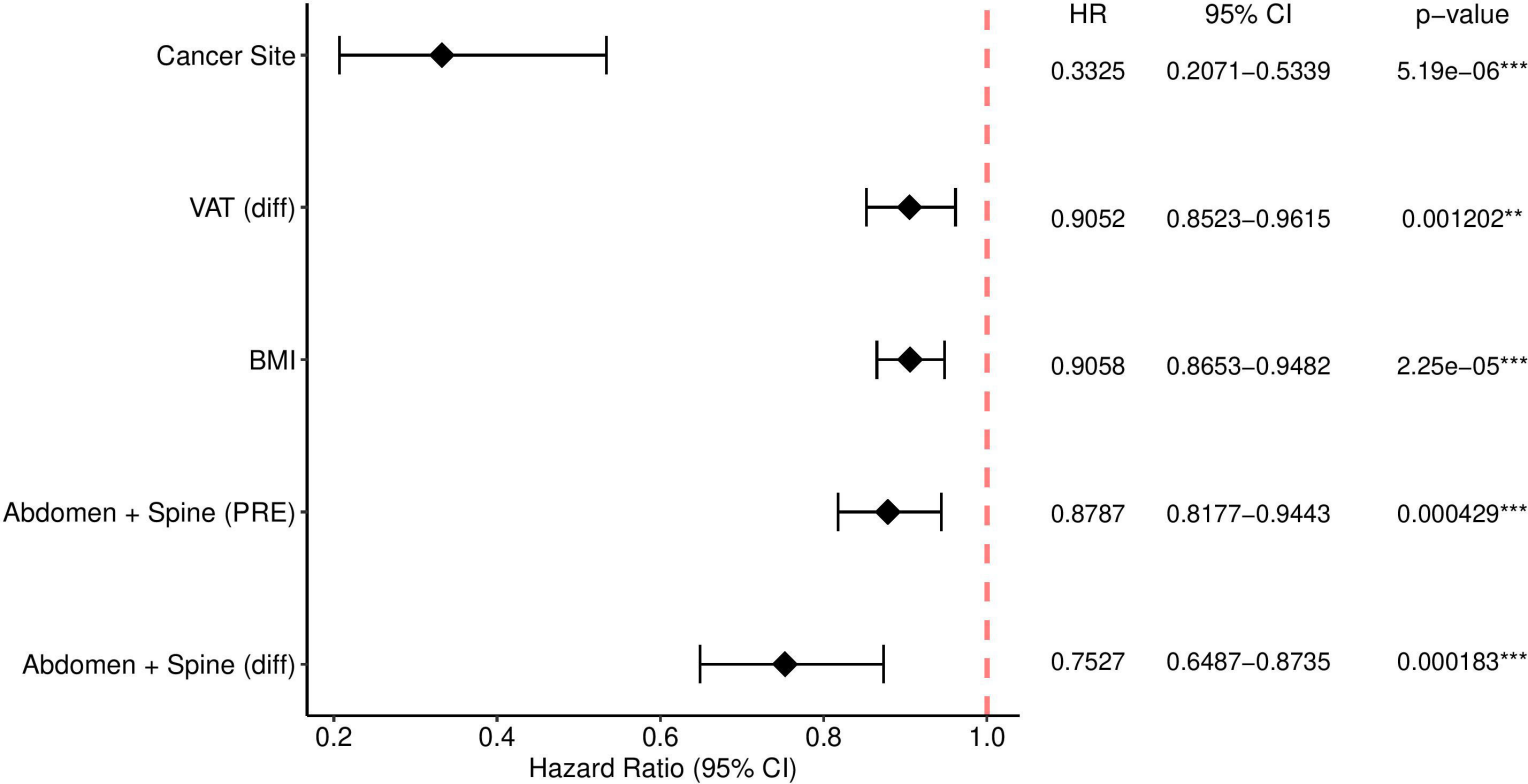
The Cox model with significance codes 0 ‘***’ 0.001 ‘**’ with e.g. “e-06” denotes 10 to the power -6.

The results for the model described in Section 2.2.2 are found in **Figure 2**. The 3D measurements (difference between Pre-RT and post-RT Abdomen and Spine Muscles percentage, the difference between Pre-RT and post-RT VAT percentage, and the sum of Pre-RT Abdomen and Spine muscles percentage) together with BMI and Cancer Site were found significant at the level of 5%. No significance for overall survival was observed for the remaining 3D body composition measurements. Manual 2D body composition measurements including L3 skeletal muscle and adiposity index pre and post treatment weren’t significant in the aspect of overall survival.

## 4 Discussion

Body composition disturbances were negatively associated with different clinical outcomes in several groups of oncological patients (6). In this study, we developed a fully automated method for the 3D quantitative analysis of body composition and showed that these measurements are significant for the overall survival of patients with HNSCC. Furthermore, they were found to be a better indicator of overall survival than commonly used metrics – such as BMI and manual cross-sectional 2D measurements of adipose and muscle tissue. In the case of all analyzed patients, radiation therapy was used, which is crucial in curative intended treatment for head and neck malignancies. It can be used as a standalone treatment or in conjunction with surgery or chemotherapy (18).

To better understand the importance of skeletal muscle in oncological disorders, we should define the term sarcopenia - a generalized skeletal muscle disorder rooted in progressive and adverse muscle changes (19). It was associated with postoperative complications, poorer survival, and chemotherapy-induced toxicity in oncological disorders (20–23). Another component of body composition analysis, adipose tissue, is a well-documented factor contributing to the development of various types of cancer (24). Higher volumes of VAT were identified as a risk factor for unsuspected pulmonary embolism in hospitalized patients with gastrointestinal cancer (25). Visceral adipose tissue modulates cellular radiosensitivity in patients with esophageal adenocarcinoma (26), it was also found that it can be considered a prognostic indicator for patients with endometrial cancer (27). Male patients with non-clear cell renal carcinoma have a higher content of visceral adipose tissue (28).

Both sarcopenia and visceral adipose tissue volume and CT attenuation were associated with the outcomes of patients with head and neck cancer (29, 30). Radiologically measured body composition parameters commonly require abdominal CT, which in the case of a patient with HNSCC is available as part of a whole-body PET/CT scan. Manual segmentation of muscles and adipose tissue is very tedious; therefore, most researchers only use a single slice from the L3 level for BC evaluation. Although it can be an accurate method to assess sarcopenia and body composition (31), this is only an approximation that can vary between patients (32), in our study the indices calculated from manually determined 2D cross sectional areas were not found significant, contrary to our automated 3D measurements. However, previous studies conducted on different groups of patients with HNSCC showed that measurements at the level of L3 can also be associated with survival (33) similar to those taken from the neck region (34–36). Nevertheless, the more cross-section slices we use, the more accurate body composition analysis will be. The efficacy of our method hinges on precise segmentation of tissues within a volumetric body region confined between L1 and L5 levels. Nevertheless, due to considerable variability in patient anatomy, accurately segmenting tissues can pose challenges in certain cases. In order to address the influence of patient anatomy variability, we leveraged a dataset consisting of 560 single-channel CT slice examinations encompassing a wide spectrum of anatomical differences. This dataset encompassed patients with diverse body sizes and shapes. Despite the model robustness, clinical expertise remains paramount in complementing and validating the model output, safeguarding the highest standard of patient care and diagnostic accuracy.

Due to the rapid progress in the field of Artificial Intelligence, segmentation of medical images, which as stated before is crucial for BC analysis, is becoming less time-consuming and more accurate. Although there are several ways for achieving automated segmentation of tissues e.g., non-local means and morphological operations (37) we decided to utilize U-net model for automatic segmentation due to the large variability in the distribution of muscle and adipose tissue. Similar approach was used for different tissues with high variability e.g., prostate gland and its zones (38). Currently, there are already a few published studies describing methods for fully automated 3D BC analysis (15, 32), but to our knowledge, we conducted the only study in which these measurements were related to survival in HNSCC patients’ group.

AI rapid advancement is also intricately tied to the field of Radiomics, an emerging discipline focused on medical image analysis which provides ways for quality assessment of different tissues by employing advanced mathematical techniques to extract additional data, probing the nuanced spatial distribution of signal intensities and pixel interrelationships (39).Together with hybrid machine learning systems it was already used in several clinical settings such as prediction of cognitive decline in Parkinson’s disease (40) or prediction of progression-free survival outcome in head and neck cancer patients (41). Although, its potential in body composition studies hasn’t been widely studied, there are already few published papers describing its usage in prediction of survival in oncological patients (42–47).

This study has several limitations. In the case of study design, its retrospective nature must be considered because it comes with certain standard disadvantages. Additionally, although PET/CT is often used in current diagnosis schemas for head and neck cancer (48), not all patients in every clinical center have done it before and after radiation therapy treatment. In the case of body composition analysis, we only used quantitative measurements of muscles and adipose tissue, which is insufficient to fully describe their characteristics but was enough to find a correlation with overall survival.

Future studies should take into consideration not only the quantity of muscles and adipose tissue but also quality, furthermore, PET/CT is not only limited to the abdominal region so it can be used for body composition measurement from more regions which could lead to some new insights. Additionally, utilizing radiomic features derived from 3D body composition data alongside advanced machine learning algorithms can unlock deeper insights into predictive models for survival. This approach may uncover subtle patterns that are not readily apparent through visual inspection. While our initial study focuses on HNSCC patients, the methodology itself is not restricted to this specific population. By encouraging further research and collaboration to assess the generalizability of our approach on diverse patient cohorts, our study aims to contribute to the development of AI-based tools that can be applied effectively and safely in a broader range of clinical settings and patient populations.

## 5 Conclusion

Body composition was previously associated with the clinical results of oncological patients. In this study we presented that 3D body composition measurements for L1-L5 levels, acquired automatically using the deep neural network U-net model implemented in the nnUnet framework, have a significant impact on the overall survival time of patients with head and neck squamous cell carcinoma. Moreover, we found out that Fully Automated 3D quantitative measurements of body composition are a better indicator of overall survival than BMI and manual cross-sectional 2D measurements of adipose and muscle tissue. To fully describe the characteristics of body composition of HNSCC patients, further research that incorporates qualitative analysis of muscle and adipose tissue, is needed.

## Data availability statement

Publicly available datasets were analyzed in this study. This data can be found here: https://wiki.cancerimagingarchive.net/pages/viewpage.action?pageId=24281354#242813540dfd743a8c3341f482a1cc44952110ec.

## Ethics statement

The studies involving humans were approved by dataset source institution - University of Texas MD Anderson Cancer Center. Our research was a retrospective study of publicity available fully anonymized imaging and clinical data. The studies were conducted in accordance with the local legislation and institutional requirements. The participants provided their written informed consent to participate in this study.

## Author contributions

MR: Conceptualization, Data curation, Investigation, Methodology, Project administration, Resources, Visualization, Writing-original draft, Writing-review & editing. DG: Conceptualization, Data curation, Formal analysis, Investigation, Methodology, Resources, Software, Visualization, Writing-review & editing. IK: Data curation, Investigation, Methodology, Resources, Writing-review & editing. ES-K: Data curation, Formal analysis, Software, Visualization, Writing-original draft, Writing-review & editing. ZT: Conceptualization, Data curation, Formal analysis, Investigation, Methodology, Resources, Software, Supervision, Visualization, Writing-original draft, Writing-review & editing. AU: Investigation, Supervision, Writing-review & editing. SK: Investigation, Supervision, Writing-review & editing. WW: Conceptualization, Investigation, Methodology, Resources, Supervision, Visualization, Writing-original draft, Writing-review & editing. All authors contributed to the article and approved the submitted version.
